# Which patient and treatment factors are related to successful cardiovascular risk score reduction in general practice? Results from a randomized controlled trial

**DOI:** 10.1186/1471-2296-14-123

**Published:** 2013-08-23

**Authors:** Ans H Tiessen, Andries J Smit, Jan Broer, Klaas H Groenier, Klaas Van der Meer

**Affiliations:** 1 University of Groningen, University Medical Center Groningen, Department General Practice, Groningen, the Netherlands; 2 University of Groningen, University Medical Center Groningen, Department Internal Medicine, Groningen, the Netherlands; 3 Municipal Public Health Service, Groningen, the Netherlands

**Keywords:** Primary health care, Arteriosclerosis, Cardiovascular diseases, Prevention and control, Self-management, Risk factors

## Abstract

**Background:**

Cardiovascular disease is a leading cause of death. It is important to identify patient and treatment factors that are related to successful cardiovascular risk reduction in general practice. This study investigates which patient and treatment factors are related to changes in cardiovascular risk estimation, expressed as the Systematic Coronary Risk Evaluation (SCORE) 10 year risk of cardiovascular mortality.

**Methods:**

179 general practice patients with mild-moderately elevated cardiovascular risk followed a one-year programme which included structured lifestyle and medication treatment by practice nurses, with or without additional self-monitoring. From the patient and treatment data collected as part of the “Self-monitoring and Prevention of RIsk factors by Nurse practitioners in the region of Groningen” randomized controlled trial (SPRING-RCT), the contribution of patient and treatment factors to the change in SCORE was analysed with univariate and multivariate analyses.

**Results:**

In multivariate analyses with multiple patient and treatment factors, only SCORE at baseline, and addition of or dose change in lipid lowering or antihypertensive medications over the course of the study were significantly related to change in SCORE.

**Conclusions:**

Our analyses support the targeting of treatment at individuals with a high SCORE at presentation. Lipid lowering medication was added or changed in only 12% of participants, but nevertheless was significantly related to ΔSCORE in this study population. Due to the effect of medication in this practice-based project, the possible additional effect of the home monitoring devices, especially for individuals with no indication for medication, may have been overshadowed.

**Trial registration:**

trialregister.nl NTR2188

## Background

**Figure 1:**
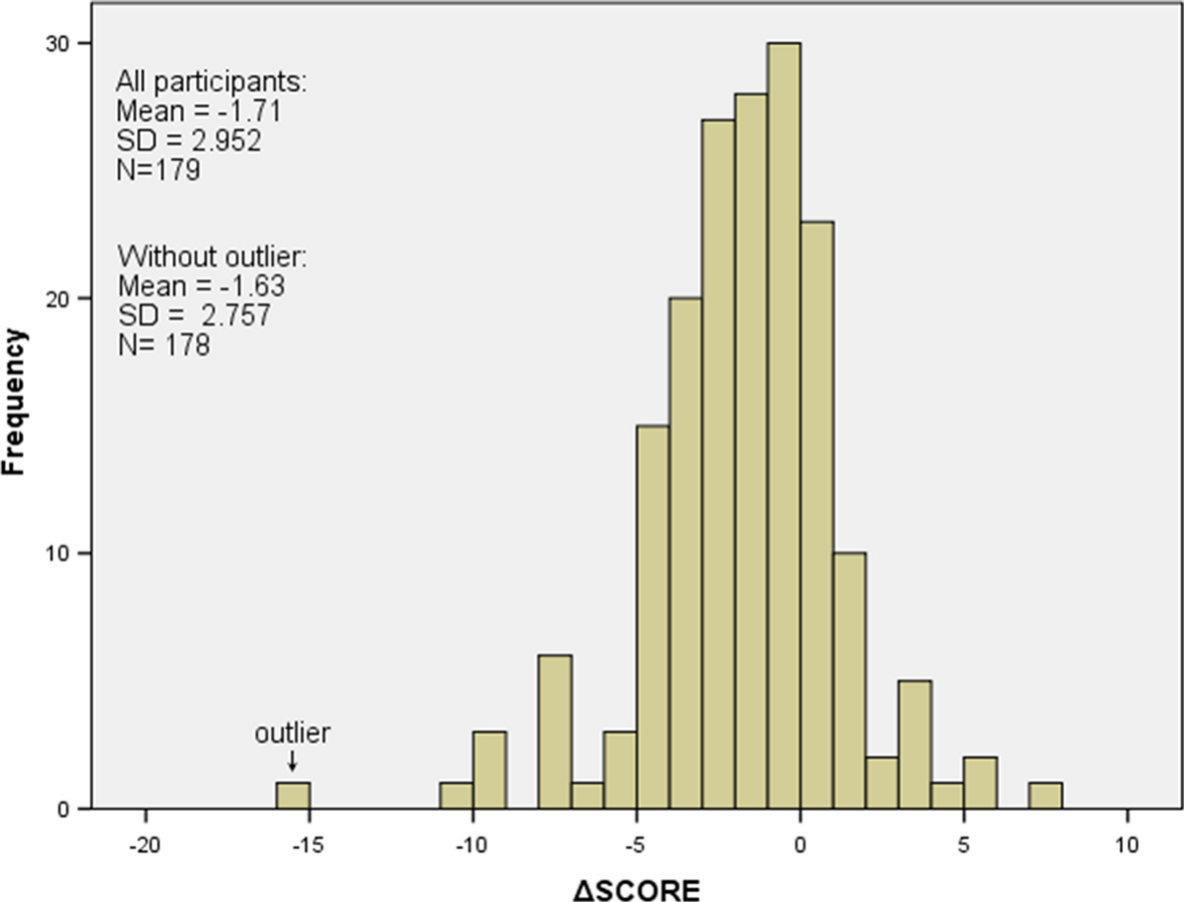
**Distribution of ΔSCORE for all participants after one year of cardiovascular risk management (a negative number for ΔSCORE means that the estimated 10-year cardiovascular risk decreased after one year).** The outlier was not included in subsequent analyses.

Cardiovascular disease (CVD) is a leading cause of death both worldwide (29% of all deaths, 2004) [1] and in the Netherlands (28% of all deaths, 2011; Statistics Netherlands). The previously reported “Self-monitoring and Prevention of RIsk factors by Nurse practitioners in the region of Groningen” randomized controlled trial (SPRING-RCT) assessed Systematic Coronary Risk Evaluation (SCORE) 10 year risk of cardiovascular mortality in individuals with a mild to moderately elevated cardiovascular risk [2,3]. This study demonstrated that one year of combined lifestyle and medication treatments by practice nurses led to a significant decrease in cardiovascular risk (Figure 1) and showed that this effect was not increased with self-monitoring.

As treatment goals for cardiovascular risk factors are often not achieved, [4,5] it is important to identify patient and treatment factors that are related to successful cardiovascular risk reduction in clinical practice. Strategies that are generally assumed to be effective include individualized risk assessment, risk communication and goal setting, and these are typically incorporated into national and international guidelines as well as research projects [6-8]. These elements formed part of the treatment in both study groups of the SPRING-RCT. The main difference between the treatment groups in this study was the use of self-monitoring as a basis for feedback and counselling (intervention group). In addition, previous analysis of this data has revealed that total consultation time and the use of antihypertensive medication were higher in the intervention group [3].

The objective of this paper is to investigate which patient and treatment factors are related to changes in cardiovascular risk estimation in individuals with a mild to moderately elevated cardiovascular risk and who are enrolled in a programme of structured lifestyle and medication counselling with or without self-monitoring.

## Methods

For the purposes of this investigation, patient and treatment data collected as part of the SPRING-RCT were further analysed. Between June 2008 and August 2009 randomly selected individuals from 20 general practices were invited for a screening. Men aged 50–75 years and women aged 55–75 years were enrolled in the study if they met the following criteria: 1) Estimated SCORE 10-year risk of cardiovascular mortality ≥5% [2]; 2) at least one treatable cardiovascular risk factor (smoking, hypertension, lack of physical activity or overweight); and, 3) no history of CVD, diabetes mellitus, thyroid dysfunction or an estimated life expectancy <2 years. Patients were randomised into control and intervention groups. All patients received standard treatment according to the 2006 Dutch general practitioner’s guidelines, [6] from specially trained practice nurses. The intervention group additionally received counselling based on self-monitoring at home with pedometers, weighing scales and/or blood pressure monitoring devices. After one year data from 179 participants were collected and analysed. The primary outcome was the SCORE 10-year risk of fatal CVD which is based on sex, age, blood pressure, lipid levels and smoking status and mean change in this did not differ significantly between groups (control group −1,63%; intervention group −1,79%). The SPRING study was approved by the Medical Ethics Review Committee of the University Medical Centre Groningen (reference number 2007/232). For more detailed information on study design and outcomes, see Tiessen et al. [3].

### Statistical analysis

General Linear Modelling was used for univariate (individual patient and treatment factors as independent variables) and multivariate (all patient and treatment factors as independent variables) analyses, with the ΔSCORE as the dependent variable. For a description of all analysed variables see Table 1. Figure 1 shows one outlier with respect to the distribution of the ΔSCORE, which was removed from further analyses. All variables from the univariate analyses were included in the multivariate model (not only the significant ones), in order to determine which factors contribute to the ΔSCORE and to what extent. In addition to investigating the effect of consultation time on ΔSCORE, we analysed the relative contribution of consultation time and different factors, which are: 1) the self-monitoring devices (home blood pressure device, home weighing scale and home pedometer); 2) more than one visit for smoking cessation; and 3) treatment intensity (percentage of indicated treatment goals for which treatment had actually been started) on ΔSCORE. Data from participants with missing data values were compared with data from participants with complete data sets and plots of residual vs. predicted values and QQ plots of the residuals were visually analysed to check the assumptions on normal distribution. A p-value <0.05 was considered significant. We used IBM SPSS statistical software version 20.

**Table 1:** Description and explanation of variables included in the analysis

Variable*	**Explanation**
Dependent variable:	
ΔSCORE (%)	Estimated 10-year cardiovascular mortality risk according to SCORE** after one year minus estimated 10-year cardiovascular mortality risk at baseline (negative numbers indicate a decrease in estimated risk)
Independent variables:	
Patient characteristics:	
Sex (male/female) n (%) male	Sex of participant
Age (years) Mean (SD)	Age of participant
Level of education (4 levels) n (%)	Self-reported highest level of education of participant
SCORE at baseline (%) Mean (SD)	Estimated 10-year cardiovascular mortality risk according to SCORE** at baseline of participant
Treatment characteristics:	
Treatment group (control/intervention) n (%) interv.	Treatment group into which participant was randomised: with (intervention group) or without (control group) self-monitoring
Consultation time (minutes) Mean (SD)	Total duration (in minutes) of all visits to practice nurse in the framework of the SPRING study
Treatment intensity (%) Mean (SD)	% of indicated treatment goals for which treatment had actually been started
Home blood pressure device	Was a home blood pressure device used as part of the SPRING study?
Home weighing scale	Was a home weighing scale used as part of the SPRING study?
Home pedometer	Was a home pedometer used as part of the SPRING study?
More than one visit for smoking	Was smoking cessation discussed at more than one visit to the practice nurse?
New/changed medication for cholesterol	Was medication for cholesterol newly prescribed or the dosage altered?
New/changed medication for hypertension	Was medication for hypertension newly prescribed or the dosage altered?

*The independent variables are described in the following manner: variable (description of categories/unit of measurement) n (%) category/Mean (SD), except for the variables with categories yes/no: these are not described any further and the given results are n (%) of “yes”.

**adapted Systematic Coronary Risk Evaluation, as used in the 2006 Dutch General Practitioner’s Guideline on cardiovascular risk management.

## Results

Total consultation time (i.e. total number of minutes participants spent visiting the practice nurse) was significantly related to a decrease in SCORE in the univariate analysis (Table 2): The value of B=−0.012 (95% CI −0.018, -0.005) corresponds to a decrease in SCORE of 0.012% with every extra minute of consultation time over the course of the study. The treatment intensity, more than one visit for smoking cessation and the use of the self-monitoring devices (except the pedometer) were also significantly related to a decrease in SCORE in the univariate analyses (Table 1). However, when these factors were analysed together, only the use of the self-monitoring blood pressure device was significantly related to a decrease in SCORE (B=−1.188 (95% CI −2.186, -0.191)).

**Table 2:** The relationship between factors affecting consultation time and ΔSCORE

	**n (%) unless otherwise indicated**	**n missing**	**Univariate analysis**			**Multivariate analysis**	
			**B**	**95% CI**	**R**^**2 **^**(adj. R**^**2**^**)**	**B**	**95% CI**
Consultation time (minutes) Mean (SD)	97 (64)	3	−0.012	−0.018, −0.005*	0.074 (0.069)	−0.005	−0.014, 0.004
Treatment intensity (%) Mean (SD)	79 (24)	1	−0.024	−0.040, −0.007*	0.043 (0.037)	−0.018	−0.035, 0.000
Home blood pressure device	43 (24)	0	−1.557	−2.484, −0.630*	0.059 (0.053)	−1.188	−2.186, −0.191*
Home weighing scale	42 (24)	0	−1.238	−2.183, −0.292*	0.037 (0.031)	−0.630	−1.874, 0.614
Home pedometer	30 (17)	0	−0.940	−2.024, 0.144	0.016 (0.011)	0.647	−0.936, 2.230
More than one visit for smoking	27 (15)	0	−1.231	−2.356, −0.105*	0.026 (0.020)	−0.998	−2.121, 0.126

95% CI= 95% confidence interval of B, R^2^ = explained variance, adj.= adjusted. *= significant, Multivariate analysis R^2^= 0.130 (adjusted R^2^= 0.099).

In this study the only patient factor shown to be significantly related to a decrease in SCORE in both uni- and multi-variate analysis (multivariate analysis, B=−0.246 (95% CI −0.363, -0.128)) was the SCORE at baseline (Table 3). All studied treatment factors were significantly related to a decrease in SCORE in the univariate analysis, except treatment group and pedometer. In the multivariate analysis with all patient en treatment factors, only added/changed medication for hypertension and cholesterol remain significant, B= −1.051 (95% CI −2.039, -0.062) and B= −2.067 (95% CI −3.247, -0.886), respectively.

**Table 3:** Patient and treatment characteristics and their relationship to ΔSCORE

	**n (%) unless otherwise indicated**	**n missing**	**Univariate analysis**			**Multivariate analysis**	
			**B**	**95% CI**	**R**^**2 **^**(adj. R**^**2**^**)**	**B**	**95% CI**
Patient characteristics:							
Sex (male/female) n (%) male	123 (69)	0	0.246	−0.638, 1.131	0.002 (−0.004)	0.666	−0.349, 1.681
Age (years) Mean (SD)	65 (5)	0	0.012	−0.063, 0.087	0.001 (−0.005)	0.038	−0.041, 0.118
Level of education (4 levels) n (%):		2			0.015 (−0.003)		
1: No education or only primary education**	17 (10)						
2: Lower secondary education	74 (42)		1.012	−0.458, 2.482		0.322	−1.003, 1.648
3: Higher secondary education	47 (26)		1.183	−0.363, 2.730		0.621	−0.812, 2.055
4: College or university	38 (21)		1.157	−0.438, 2.751		−0.213	−1.695, 1.270
SCORE at baseline (%) Mean (SD)	8.6 (4.1)	0	−0.226	−0.321, −0.130*	0.111 (0.105)	−0.246	−0.363, −0.128*
Treatment characteristics:							
Treatment group (control/intervention) n (%) interv.	89 (50)	0	−0.387	−1.203, 0.429	0.005 (−0.001)	0.722	−0.223, 1.667
Consultation time (minutes) Mean (SD)	97 (64)	3	−0.012	−0.018, −0.005*	0.074 (0.069)	0.000	−0.009, 0.009
Treatment intensity (%) Mean (SD)	79 (24)	1	−0.024	−0.040, −0.007*	0.043 (0.037)	−0.009	−0.026, 0.008
Home blood pressure device	43 (24)	0	−1.557	−2.484, −0.630*	0.059 (0.053)	−0.489	−1.474, 0.496
Home weighing scale	42 (24)	0	−1.238	−2.183, −0.292*	0.037 (0.031)	−1.138	−2.356, 0.080
Home pedometer	30 (17)	0	−0.940	−2.024, 0.144	0.016 (0.011)	0.115	−1.345, 1.576
More than one visit for smoking	27 (15)	0	−1.231	−2.356, −0.105*	0.026 (0.020)	−0.644	−1.712, 0.424
New/changed medication for cholesterol	22 (12)	0	−3.426	−4.559, −2.292*	0.168 (0.163)	−2.067	−3.247, −0.886*
New/changed medication for hypertension	46 (26)	0	−2.195	−3.071, −1.320*	0.122 (0.117)	−1.051	−2.039, −0.062*

95% CI= 95% confidence interval of B, R^2^ = explained variance, adj.=adjusted. *= significant; **=reference category, Multivariate analysis R^2^= 0.360 (adjusted R^2^= 0.298).

There were very few missing data values and only age was significantly different between participants with and without missing values, median age 69.5 years and 65.0 years for participants with and without missing values, respectively (p=0.02, Mann–Whitney *U* test). No abnormalities were found with respect to the assumptions.

## Discussion

### Summary of main findings

The goal of this paper was to investigate which patient and treatment factors are related to changes in cardiovascular risk estimation. Our results show that in the univariate analysis, the consultation time is significantly related to the ΔSCORE. However, when the analysis includes the use of separate devices and treatment factors, only the use of the home blood pressure device was significantly related to the ΔSCORE, and consultation time was not significant anymore. The analyses with all studied patient- and treatment factors show that ΔSCORE is related to both baseline SCORE and changed/added antihypertensive and lipid lowering medication in the multivariate analysis.

### Comparison with existing literature

Diabetes prevention and smoking cessation studies have demonstrated that success is more likely in patients who are closely monitored [9,10]. It is therefore possible that a similar positive effect may be seen in this study; namely a reduction in ΔSCORE in the patients using self-monitoring devices and being, therefore, potentially more intense follow-up with more consultation time. However, the significant effect of consultation time on ΔSCORE is not shown in our multivariate results.

A possible explanation for the influence of the use of the home blood pressure device on ΔSCORE (Table 2) is that this is the only self-monitoring device used in this study that is directly related to one of the components of SCORE risk estimation. In contrast, use of the weighing scale and pedometer for the monitoring of weight and physical activity are not part of the SCORE calculation. Furthermore, the home blood pressure device and the home weighing scale were each used by a large number of participants (24%) whereas the pedometer was only used by 17% of the participants and is therefore unlikely to have a significant effect on the total ΔSCORE of all participants. The failure to show a significant relationship between ΔSCORE and the number of smoking-related visits made during the study despite the direct effect of this variable on a risk factor included in the SCORE calculation could similarly be explained by the small fraction of subjects in this category (15%). In this study treatment intensity, which may reflect the motivation of the patient was not significantly related to the overall decrease in SCORE seen in the multivariate analysis.

The second part about comparison with existing literature focusses on the analyses with all studied patient and treatment factors. These analyses show that ΔSCORE is related to both baseline SCORE and changed/added antihypertensive and lipid lowering medication in the multivariate analysis, with an explained variance of 36% (Table 3). This effect was not related to the fact that only participants with a substantially elevated cardiovascular risk were advised to take medication, as both medication data and baseline SCORE were independently related to ΔSCORE. The positive effect of lipid lowering medication on cardiovascular risk reported here is particularly remarkable given that only 12% of patients were newly prescribed with or received an altered dose of lipid lowering medication over the course of this study. The effect of anti-hypertensives on cardiovascular risk is less surprising, because 26% of participants received a new prescription or altered dose of this medicine during the study. These findings combined with the relatively low cost of the medications, [11] serve to confirm the important role of statins and antihypertensives as part of a cardiovascular risk reduction programme [12,13].

We expected that participants with hypertension would receive either home monitoring as well as (added/changed antihypertensive) medication or only medication in the intervention and control groups, respectively, and that home blood pressure monitoring would not be used in patients who were not receiving medication. Our results show an unexpectedly high rate of home blood pressure monitoring in patients who were not receiving added/changed antihypertensive medication: Of the 178 participants included in this analysis 27 had used both medication and home monitoring for their blood pressure, 19 only medication, 16 only home monitoring and 116 made use of neither of these. This may reflect the individualized nature of the therapy in this practice-based RCT and may have influenced the relation between home blood pressure monitoring and ΔSCORE. Combined with the fact that treatment for hypercholesterolemia was the same in both control and intervention groups, the use of home blood pressure monitoring by patients in both groups may go some way to explaining the lack of significant difference between groups in terms of ΔSCORE.

With respect to the SCORE at baseline the European Guidelines on CVD prevention in clinical practice state that “the higher the risk the greater the benefit from preventive efforts” [7]. Targeting high risk individuals is also recommended by the Cochrane review on multiple risk factor interventions for primary prevention of coronary heart disease [14]. Our analyses confirm that the main focus of cardiovascular risk reduction should be the population with the highest risk levels. However, the magnitude of the effect in these participants in our study may be partly due to the regression to the mean-effect.

### Strengths and limitations

Because we intended to analyse which factors are related to ΔSCORE to what extent, and not to predict ΔSCORE, we included all variables from the univariate analyses in the multivariate model and not only the ones found to be significant with univariate analysis. Our results should not be interpreted as a clinical decision rule, but might help clinicians substantiate the focus of cardiovascular risk management at their practices [15].

The current study took place in the practice setting meaning that the results can be easily translated into general practice treatment strategies. The sample population, with a mild-moderately elevated cardiovascular risk, is a heterogeneous group with respect to individual risk factors, treatment goals and therapy chosen by each participant in cooperation with his/her practice nurse.

Most known factors influencing CVD risk were collected for the SPRING-RCT and included in the analyses. Whilst it has been shown that psychological factors such as stress, motivation, self-efficacy and received help may have an impact on cardiovascular risk and risk factor reduction, these aspects were not assessed in this study [10,16]. All studied variables were present in a considerable proportion in the study population, as can be seen in Tables 2 and 3. However, as the data were collected as part of the SPRING-RCT, the sample size was calculated for the primary outcome of this RCT and not for the analyses of this paper. Finally, the research staff who did the measurements at follow-up was not blinded. However, SCORE was not directly measured, so this influence might be limited.

Interpretation of our results is facilitated by the use of unstandardized coefficients and their 95% confidence intervals which clearly show the size of the effect on the ΔSCORE, given a certain value of the specific independent variable.

We did not collect data on long term outcomes regarding cardiovascular morbidity and mortality, because our endpoint is the ΔSCORE where SCORE is a tool for risk stratification. The adapted SCORE, based on the Dutch situation, which was used in the 2006 Dutch General Practitioner’s Guideline on cardiovascular risk management as well as in this study, appears to overestimate cardiovascular mortality [17]. This is possibly due to the decline in cardiovascular mortality. This overestimation was reported in many European countries [18-21] but not in all [22,23]. The significant effect of medication on the ΔSCORE probably reflects the direct effect of lipid levels and blood pressure on the parameters included in the calculation of SCORE. Because of this, it is important that the ΔSCORE reported here should not be considered as an exact predictor of decreased mortality risk, but as an indicator for cardiovascular risk reduction.

## Conclusions

For successful cardiovascular risk management, defined as greatest feasible reduction in the ΔSCORE at general practice level, it is clear that treatment should be focused at individuals with high SCORE at presentation. Medication (lipid lowering and antihypertensive) was independently related to a significant decrease in estimated cardiovascular risk, even though lipid lowering medication was added/changed in only 12% of the participants. It is also worthy of note that lipid lowering medication was the only intervention factor for which no intensive/self-monitoring treatment alternative was offered in the intervention group. The SPRING-RCT was set up to investigate whether counselling based on home monitoring would have an additional effect on the estimated cardiovascular risk. Due to the effect of medication in this practice-based project, the possible additional effect of the home monitoring devices, especially for individuals with no indication for medication, may have been overshadowed. However, based on these results we recommend that for optimal risk reduction in general practice treatments (including prescription of medications when indicated and agreed with by the patient) for cardiovascular risk be targeted at high-risk individuals.

## Ethics approval

The SPRING study was approved by the Medical Ethics Review Committee of the University Medical Centre Groningen: 2007/232.

## Abbreviations

SCORE: Systematic coronary risk evaluation; SPRING: Self-monitoring and prevention of risk factors by Nurse practitioners in the region of Groningen; RCT: Randomized controlled trial; CVD: Cardiovascular disease; GP: General practice/practitioner.

## Competing interests

The authors declare that they no competing interests.

## Author’s contributions

AS, JB, KM and AT designed the study, AT collected the data, AT and KG analysed the data, AT drafted the article, all other authors offered revisions and gave final approval of the submitted version. All authors read and approved the final manuscript.

## Pre-publication history

The pre-publication history for this paper can be accessed here:

http://www.biomedcentral.com/1471-2296/14/123/prepub
